# Effect of COVID-19 on Blood Pressure Profile and Oxygen Pulse during and after the Cardiopulmonary Exercise Test in Healthy Adults

**DOI:** 10.3390/jcm12134483

**Published:** 2023-07-04

**Authors:** Kamila Miętkiewska-Szwacka, Remigiusz Domin, Małgorzata Kwissa, Mikołaj Żołyński, Jan Niziński, Elżbieta Turska, Maciej Cymerys

**Affiliations:** 1 Department of Internal Medicine, Poznan University of Medical Sciences, 60-786 Poznan, Poland; kwissamalgorzata@gmail.com (M.K.); maciejcymerys@ump.edu.pl (M.C.); 2 University Centre for Sports and Medical Studies, Poznan University of Medical Sciences, 60-802 Poznan, Poland; remigiusz.domin@gmail.com (R.D.); mik.zolynski@gmail.com (M.Ż.);; 3 Department of Endocrinology, Metabolism and Internal Medicine, Poznan University of Medical Sciences, 60-355 Poznan, Poland; 4 Department of Cardiology—Intensive Therapy, Poznan University of Medical Sciences, 60-355 Poznan, Poland; 5 Institute of Pedagogy, University of Zielona Gora, 65-417 Zielona Gora, Poland

**Keywords:** blood pressure, oxygen pulse, COVID-19, cardiopulmonary exercise test, post-exercise recovery

## Abstract

Several reports have shown the impact of COVID-19 history on exercise capacity. This study compared the blood pressure (BP) response and oxygen pulse (O_2_ pulse) characteristics in normotensive patients with and without a history of COVID-19 during the cardiopulmonary exercise test (CPET) and post-exercise recovery. This cross-sectional study involved 130 healthy Caucasian adult volunteers (71 participants with a history of COVID-19). All patients underwent the CPET with blood pressure measurements during exercise and post-exercise recovery. The post-COVID group had significantly higher systolic, diastolic, and mean blood pressure after 9 min of recovery and achieved a significantly lower max O_2_ pulse (2.02 mL/beat on average) than the controls. It should be noted that the COVID group tended to have higher blood pressure values in all steps, with no differences in heart rate, pulse pressure, and saturation at any step. The COVID-19 outbreak was associated with a higher blood pressure response, significantly, in post-exercise recovery, a lower maximum O_2_ pulse, and a lower maximum load achievement. Future studies are needed to determine if these abnormalities during the CPET and the blood pressure variation have prognostic value.

## 1. Introduction

The coronavirus disease (COVID-19) pandemic, caused by the severe acute respiratory syndrome coronavirus-2 (SARS-CoV-2), is still a significant public health challenge worldwide [1,2]. COVID-19 affects different systems, particularly, the respiratory and cardiovascular ones, and is associated with clinical outcomes and various comorbidities [3,4]. About 80% of COVID-19 cases are paucisymptomatic and mild, with most patients recovering within 2–4 weeks [5]. Abnormal clinical parameters persisting for two or more weeks after COVID-19 onset that do not return to baseline can potentially be considered long-term effects of the disease. Indeed, such outcomes have been reported in patients with a mild infection who did not require hospitalization [6,7,8].

Cardiopulmonary exercise testing (CPET) is the gold standard of aerobic exercise tests and, combined with blood pressure (BP) monitoring, is one of the most effective noninvasive methods for evaluating potential cardiovascular, ventilatory, and musculoskeletal limitations during exercise for COVID-19 survivors [9,10].

Recent studies reported pre-existing hypertension as the most common COVID-19 comorbidity [11]. Pooled analyses suggested that hypertension may be associated with a higher risk of severe or fatal COVID-19, especially in older patients [12]. Moreover, the prognosis for people with hypertension is markedly worse when COVID-19 infection is complicated by cardiovascular disease and end-organ damage, associated with poorer control of high BP and mean BP rises with age [13,14,15]. However, the effect of COVID-19 on the BP response during exercise in normotensive individuals has not been investigated. The BP response during post-exercise recovery can provide relevant clinical information such as predictors of future hypertension [16].

The evaluation of exercise capacity is possible with CPET by assessing a concise overview of the cardiovascular, ventilatory, and gas exchange parameters [17]. In turn, some of these derivatives represent the integration of both respiratory and cardiovascular systems, i.e., the oxygen uptake (VO_2_) to heart rate (HR) ratio provides the O_2_ pulse (VO_2_/HR = O_2_ pulse) reflecting the amount of oxygen extracted by the tissues per heartbeat, which is an indirect index of stroke volume [18]. Differences in O_2_ pulse between healthy controls and COVID-19 survivors might reflect the effect of the disease on cardiorespiratory fitness.

This study analyzed and compared BP response and O_2_ pulse characteristics measured during the CPET to exhaustion and post-exercise recovery in normotensive and, so far, healthy people with and without a history of COVID-19.

## 2. Materials and Methods

### 2.1. Study Design and Test Participants

The study population comprised 130 healthy Caucasian participants (69 men and 61 women) aged 18–66 years. The inclusion criteria were: (1) SARS-CoV-2 infection not requiring hospitalization or home oxygen therapy and (2) a minimum of one month of recovery from COVID-19. The exclusion criteria were: (1) a history of chronic disease requiring chronic pharmacotherapy (except oral hormonal contraception in women, ad hoc non-steroidal anti-inflammatory drugs, and anti-allergic drugs), (2) professional athletes, and (3) an abnormal resting electrocardiogram (ECG).

The study was conducted in accordance with the ethical principles of the Declaration of Helsinki and was approved by the Bioethical Committee at Poznan University of Medical Sciences, Poznan, Poland (approval no. 519/21 in 2021) [19]. All participants provided informed consent and agreed for their anonymized clinical and investigative data to be used for research purposes. They were instructed to wear a comfortable outfit, avoid exercise/physical labor 24 h hours before the test, fast for 3 h, and not to smoke for at least 8 h before the CPET following the Association for Respiratory Technology and Physiology Guidelines (ARTP Guidelines) [20].

### 2.2. Resting Clinical Assessment

All participants underwent a detailed clinical evaluation including: (1) medical history (the date of COVID-19 onset was recorded) and physical examination, (2) measurement of anthropometric parameters, (3) measurement of resting BP and resting HR, assessed using an automatic BP monitor Omron M7 Intelli IT (Omron, Kyoto, Japan), and (4) resting 12-lead electrocardiography (ECG), using a Mortara Wireless Acquisition Module (WAM) (Mortara Instrument INC, Milwaukee, WI, USA).

### 2.3. Exercise Testing Equipment

The CPET was performed on a specialized, electromagnetically braked cycle ergometer (Corival, Lode B.V., Groningen, The Netherlands) using a CPET system (Vyntus CPX powered by SentrySuite, Vyaire Medical, Mettawa IL, USA) with s breath-by-breath gas analyzer by experienced physicians who perform CPET regularly. The CPET system was calibrated following the manufacturer’s recommendations before each test. An integrated Nonin device was used to measure SpO_2_ with an ear sensor probe. Stress ECG and HR were monitored at rest and throughout the test using a 12-lead exercise ECG (Mortara Wireless Acquisition Module, Mortara Instrument INC, Milwaukee, WI, USA). Cuff BP was measured every 3 min during exercise and post-exercise recovery, using an adult cuff with a shockproof sphygmomanometer (Gamma G5, HEINE Optotechnik GmbH & Co. KG, Gilching, Germany).

### 2.4. Exercise Protocol and BP Measurements

Before the CPET, resting spirometry was performed using a CPET system (Vyntus CPX, Vyaire Medical, Mettawa IL, USA) according to the ARTP Guidelines [20]. Based on the physician’s experience, an incremental ramp protocol was individualized to complete the proper progressive exercise phase of the CPET optimally in 10 ± 2 min, with the median test time of 9.63 min (~580 s) reflecting adequate protocol individualization. The first protocol component was the rest phase, in which all parameters were recorded without any exercise being performed and lasted 2 min, then the 3 min warm-up phase started with the participants instructed to pedal with a constant cadence (approximately 60–90 revs/min) throughout the test. After the warm-up, the progressive exercise proceeded and continued until the participants were exhausted (refusing to pedal further) or the physician observed any abnormalities in BP, ECG, or clinically indicated symptoms (e.g., angina or dyspnea). The final phase was the registration for 15 min of post-exercise recovery without pedaling.

The physician manually measured the BP on the upper arm during the test at rest, after 3, 6, and 9 min, at maximum activity, and after 3, 6, 9, 12, and 15 min of recovery.

This paper presents the data regarding two parameters: the O_2_ pulse, which is an indirect measure of stroke volume (SV) and load (Watts). The rest of the collected data is beyond the scope of this investigation and is published in a further article.

### 2.5. Statistical Analysis

The significance level of the statistical tests was set at α = 0.05. The *p*-value was calculated using the Tukey’s correction method for multiple comparisons. The distribution of measures of central tendency for numerical variables was expressed by Mdn (Q1, Q3). For nominal variables, the distribution was determined by specifying the frequency of each category and the percentage of the total, *n* (%). The significance of the differences between the means of two independent groups for the numerical variables was determined using the Wilcoxon rank sum test and the Pearson’s chi-square test for two categorical variables.

To estimate the effects of the explanatory variables on the performance and BP parameters, eight linear mixed regression models were estimated and fitted in the form of different factorial designs (Appendix A for CPET and BP parameters) using restricted maximum likelihood (REML) and boundary optimizer based on quadratic approximation (BOBYQA). The patient’s ID was included as a separate random effect, and 95% confidence intervals (CIs) and *p*-values were computed using a Wald *t*-distribution approximation.

The statistical analyses were conducted using the R Statistical language (version 4.1.1; R Core Team, 2021) on Windows 10 Pro 64-bit (build 19044).

## 3. Results

This study involved 130 participants including healthy subjects (*n*_1_ = 59; 45.4%) and post-COVID participants (*n*_2_ = 71; 54.6%). The participants’ sociodemographic data by group are presented in Table 1, showing that the post-COVID group was significantly older than the control group. All subjects completed the exercise test without any complications, and no subjects were excluded because of poor motivation.

### 3.1. The Effects of COVID and Exercise Steps on the CPET (Models 1,2)

The estimated marginal means (EMMs) for step and group predictors in a linear model for load (Model 1) and O_2_ pulse (Model 2) are presented in Appendix B (Table A1). The results of the simple contrasts used to estimate the effects between the groups within marginal mean maximum load = 239 Watts and steps are shown in Appendix C (Table A2).

**Figure 1:**
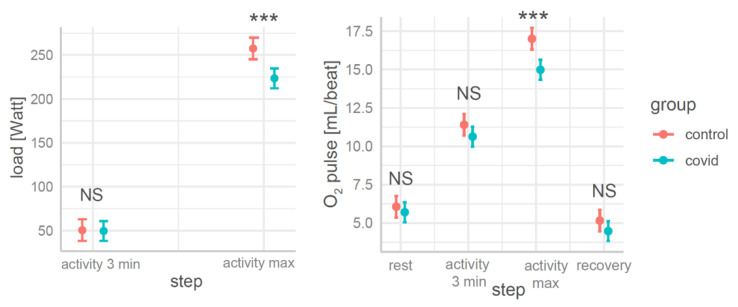
The load (**left**) and O_2_ pulse (**right**) in terms of steps and groups with significant differences between the groups for the fitted models 1,2 (NS—non-significant; ***—*p* < 0.001). Load and O_2_ pulse were significantly lower at maximal activity in the COVID group.

There were no significant differences in load (the difference was about 1 Watt) between the participants in the control and COVID groups after 3 min of activity on the cycle ergometer (Figure 1). In the activity maximum step, the load values in the control group were significantly higher (33.95 Watts higher on average) than those in the COVID group, but only the O_2_ pulse values in the COVID group in the activity maximum step were significantly lower (2.02 mL/beat on average) than in the controls (Figure 1).

### 3.2. The Effects of COVID and Exercise Steps on Blood Pressure Parameters (Models 3–8)

The EMMs for the step and group predictors in a linear model for systolic BP (SBP) (Model 3), diastolic BP (DBP) (Model 4), HR (Model 5), saturation (SpO_2_) (Model 6), pulse pressure (PP) (Model 7), and mean BP (MBP) (Model 8) are presented in Appendix B (Table A1). The results of the simple contrasts used to estimate the effects between the groups within a marginal mean maximum load = 239 Watts and during the exercise steps are shown in Appendix C (Table A2).

**Figure 2:**
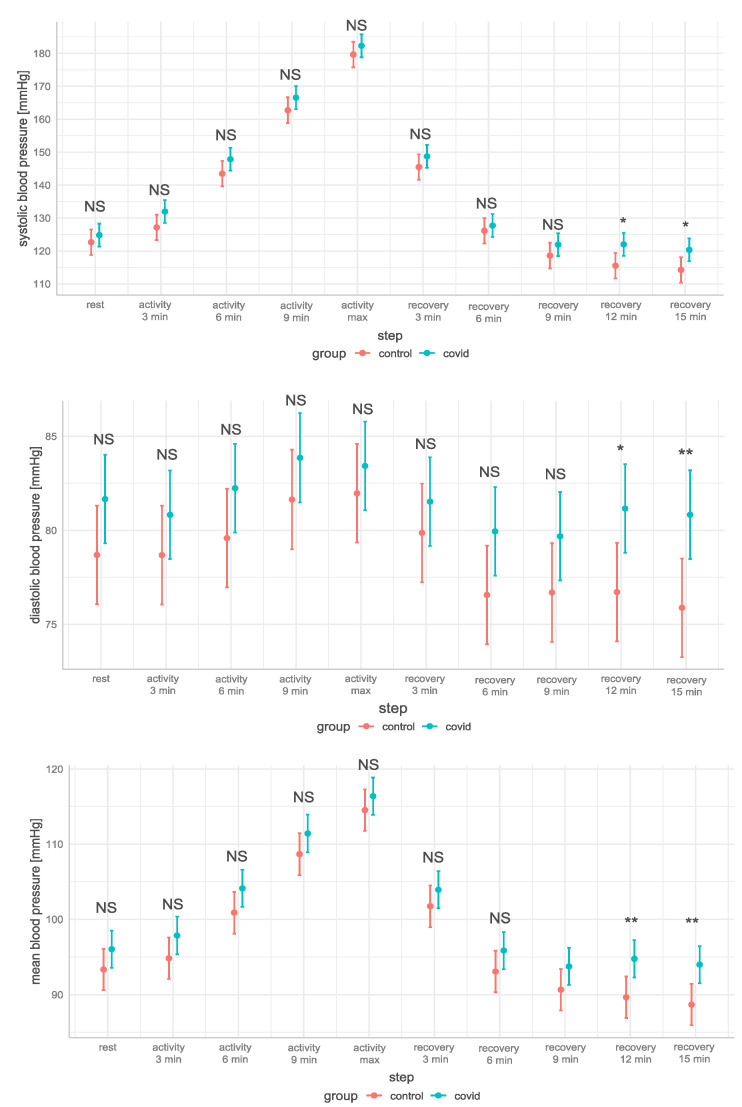
The systolic, diastolic, and mean blood pressure in terms of the marginal load max average = 239 Watts, steps, and groups, with significant differences between the groups for the fitted models 3, 4, and 8 (NS—non-significant; *—*p* < 0.05; **—0.001 ≤ *p* < 0.01). Systolic, diastolic, and mean blood pressure were significantly higher in the COVID group after 12 and 15 min of recovery.

There were no differences in SBP, DBP, and MBP in the resting phase, progressive activity phase, and initial recovery phase. However, the SBP, DBP, and MBP in the COVID group decreased significantly more slowly than in the control group after 9 min of recovery (Figure 2). It should be noted that the COVID group tended to have higher SBP, DBP, and MBP in all steps, with no differences in HR, PP and SpO_2_ at any step.

## 4. Discussion

The post-COVID participants achieved a significantly lower maximum load, a lower maximum O_2_ pulse, and a higher BP (SBP/DBP/MBP) at 12 and 15 min of post-exercise recovery, with no differences in HR, PP, and saturation at any step.

The CPET is a non-invasive method used to assess the cardiorespiratory capacity. In this study, we selected and analyzed two CPET parameters, i.e., oxygen pulse, which represents the stroke volume (SV) ratio, and O_2_ extraction from the blood per heartbeat and load (in Watts). In normal conditions, the O_2_ pulse increases during incremental-load exercise, and its increasing trend assumes the shape of a hyperbola [21]; therefore, the arteriovenous oxygen difference does not substantially change during incremental exercise, and the O_2_ pulse mainly represents the SV ratio and the left ventricular performance [22]. Flattened or reduced O_2_ pulse kinetics during exercise may reflect peripheral vascular perfusion, extraction, or central cardiogenic performance limitations [18]. Cassar et al. [23] observed a significantly lower O_2_ pulse (maximal tests) than in controls in serial CPET assessments in previously hospitalized post-COVID patients 2 to 3 months after COVID-19, which improved and became comparable to that of the controls by 6 months. Conversely, our cohort mainly consisted of mild COVID-19 cases who had not been hospitalized and had a significantly lower maximum O_2_ pulse, whose profile in all steps was flatter than that of the controls in the ~10-month follow-up. Kersten et al. [24] reported the CPET results depended on the initial disease severity about 8 months after the COVID-19 onset, whereby the non-hospitalized participants achieved a significantly lower O_2_ pulse than the hospitalized patients. Xiao et al. [25] showed that nearly half of hospitalized COVID-19 survivors did not achieve the expected O_2_ pulse values during the CPET, suggesting a relatively poor cardiac reserve. HR tends to limit the exercise capacity in healthy individuals, but in our post-COVID patients, there were no differences in HR at any stage [17]. However, we are aware that differences in O_2_ pulse are determined by VO_2_.

In healthy adults, the peak workload decreases with age (markedly over 60 years of age), and males systematically score higher than females [26]. In our study, the participants in the post-COVID group were significantly older than those in the control group, and their median age was 40 years vs. 31 years for the controls. Frizzelli et al. [27] performed the CPET after 12 months from the onset of SARS-CoV-2 infection and before recovery and reported that post-COVID-19 patients with unexplained dyspnea showed a significantly lower oxygen uptake at the peak and at the anaerobic threshold, lower maximal workload (Watts) and O_2_ pulse. Evers et al. [28] identified a reduced O_2_ pulse pattern of exercise capacity limitation and a lower workload in their double CPET post-COVID evaluation, without correlation with the initial severity of the disease, possibly due to reduced oxygen utilization and/or impaired peripheral oxygen metabolism in the absence of a macroscopic cardiocirculatory pathology. We also assume a deteriorating cardiorespiratory fitness after COVID-19, which could influence achieving the maximum workload. Back et al. [29] evaluated the CPET one month after a mild-to-moderate infection in patients without severe disease (mean age of 40 years) using a cycle ergometer, showing a significantly reduced peak workload (approximately 50 Watts) and circulatory power, as well as a lower O_2_ pulse.

Typically, SBP rises with an increasing workload (as the cardiac output increases during exercise in response to the increased demand of oxygen from the working muscles), and there is no change or a mild reduction in DBP, with a decline of both during post-exercise recovery in healthy individuals [30,31]. In our study, we focused on the variability of BP during and after the CPET in normotensives after COVID-19 using an original protocol that allowed the observation of this parameter over time, with particular emphasis on prolonging the surveillance of the post-exercise recovery phase to 15 min. Barbagelata et al. [10] performed the CPET in patients with a history of COVID-19, an average of 3 months after the viral episode, revealing a normal course of BP without an exaggerated behavior. In our study, we did not observe a hypertensive response to exercise in any group or new-onset hypertension. However, Chan et al. [32] suggested that a previous symptomatic SARS-CoV-2 infection may alter BP regulation during exercise in healthy individuals due to changes to the autonomic nervous system. In addition, Akpek et al. [33] investigated the effect of COVID-19 on BP, showing significantly higher SBP and DBP at a one-month follow-up compared to admission.

The post-exercise evaluation of BP recovery is a prognostic tool for diagnosing cardiovascular abnormalities in healthy adults undergoing exercise testing [34]. A blunted or delayed decline in SBP and an elevated SBP after exercise are also associated with an increased risk of coronary heart disease [35,36,37] or new-onset hypertension [38]. Sahrai et al. [39] reported that COVID-19 survivors may show persistently elevated BP in the convalescent period. They proposed some possible explanations for the abnormalities mentioned above: (1) activation of the RAAS or the sympathetic nervous system, (2) endothelial dysfunction, (3) delayed resolution of inflammation, or (4) hypoxia and ischemia. A meta-analysis of over 19 million people reported that post-COVID-19 individuals had an additional 70% risk of developing new-onset hypertension within 7 months of acute infection [40]. Although we could not diagnose hypertension in our post-COVID population, they tended to have higher SBP, DBP, and MBP in all exercise steps, which may be due to this cohort being relatively young (average age under 40 years) and having suffered a mild infection that did not require hospitalization.

### 4.1. Limitations

There are several limitations to this study. First, as in any cross-sectional study, the possibility of bias (mainly, a selection bias) potentially influencing the result cannot be excluded. Second, we studied adult volunteers up to 66 years of age of the Caucasian race from Poland. Therefore, our results cannot be extrapolated to children, the elderly, or other ethnic groups. Third, there was a difference between patients with and without COVID-19 at the time of enrolment. During the first COVID-19 waves, this disease was more common in older adults than in younger adults, whereas enrolment in our study was consecutive. These issues may explain the observed age difference between the two groups. Finally, we do not have data on the cardiopulmonary status of people who had COVID-19; so, the effect of any pre-existing cardiopulmonary impairment could not be fully assessed.

### 4.2. The Novelty of the Study

Some of our findings are new concerning post-COVID-19 patients. Otherwise, the healthy post-COVID-19 patients achieved lower loads (almost 34 Watts on average) and O_2_ pulse values (2.2 mL/beat on average) at the peak of exercise to exhaustion. There were no statistical differences in the BP profiles between the groups studied. However, the recovery of post-exercise BP was delayed at 12 and 15 min after exercise in people with previous COVID-19. Despite this, the average values of SBP and DBP remained within the normal limits.

## 5. Conclusions

A history of COVID-19 in otherwise healthy people is associated with reduced maximum O_2_ pulse and peak exercise load to exhaustion. In addition, these individuals showed a delayed recovery of BP after exercise compared to individuals without previous COVID-19. These findings require further clinical evaluation.

## Figures and Tables

**Table 1:** Comparison of the participants’ baseline characteristics.

Characteristic	Control, *n* = 59 ^1^	COVID, *n* = 71 ^1^	*p*-Value ^2^
sex			0.098
female	23 (39.0%)	38 (53.5%)	
male	36 (61%)	33 (46.5%)	
age	31.0 (29.0, 38.5)	40.0 (30.5, 45.0)	0.002
height, meters	1.76 (1.69, 1.82)	1.70 (1.68, 1.80)	0.216
weight, kg	76.0 (64.5, 86.0)	74.0 (63.5, 82.0)	0.568
BMI, kg/m^2^	24.22 (23.15, 25.89)	24.39 (22.46, 26.37)	0.983
smoking			0.568
yes	7 (12.0%)	6 (8.5%)	
no	52 (88.0%)	65 (91.5%)	
vaccination			0.953
yes	53.0 (89.8%)	64.0 (90.1%)	
no	6.0 (10.2%)	7 (9.9%)	
Time from COVID to CPET [years]	-	0.85 (0.50, 1.00)	-

Note: ^1^ n (%); Mdn (Q1, Q3); ^2^ Pearson’s Chi-squared test; Wilcoxon rank sum test. Abbreviations: BMI, body mass index.

## Data Availability

The datasets generated and/or analyzed for this study are currently not publicly available due to further ongoing analyses by the authors. Selected data, however, are available from the corresponding author upon request.

## Appendix A. Model Specifications

### Appendix A.1. Model 1 and 2 Studied the Effects of COVID and Exercise Steps on Performance Parameters at Rest, during Exercise on the Cycloergometer, and in the Recovery Phase

The performance parameters included load [Watt] (model 1) and O_2_ pulse [mL/beat] (model 2). The 2 × 2 factorial model design for the load dependent variable allowed the estimation of the main effects for each of two exercise steps (3 min of activity and maximal activity) for patients after COVID and the control group, along with the interaction effect (model 1). For the O_2_ pulse, the dependent variable, i.e., the exercise steps, included two additional categories (rest and recovery) resulting in a 4 × 2 factorial design (model 2).

The regression model for estimating the effects of exercise steps and group on the performance variable was specified by a multilevel variant of the simple change score model with the Formulas (A1)–(A3):

(A1) $$y_{ij}\sim N\left(\mu_{ij},\;\sigma_{\epsilon }\right)$$

(A2) $$\mu_{ij}=\beta_{0}+\beta_{1}\cdot {step}_{ij}+\beta_{2}\cdot {group}_{ij}+\beta_{3}\cdot {step}_{ij}\times {group}_{ij}+u_{0i}$$

(A3) $$u_{0i}\sim N\left(0,\;\sigma_{0}\right)$$

where the outcome variable $y_{ij}$ varies between *i* participants and *j* groups; *β*_0_ is the population mean (the model constant) in the reference category step (the 3 min activity step for the load-dependent variable, and the rest step for the O_2_ pulse - dependent variable) for the control group (baseline); *β*_1_ is the difference between the reference and other step individual categories for the control group compared with the reference step category; *β*_2_ is the change group for the reference step category; *β*_3_ is the group-by-step interaction, which is also the same as the average step effect in the population, τ.

Additionally, the model accounts for a participant-level deviation around the grand mean baseline across term *u*_0i_, which is modelled as normally distributed with a mean of zero and a standard deviation *σ*_0_.

The interaction effects were examined using a simple contrast analysis as group differences in estimated marginal means (EMMs) (predictions from a linear model over a reference grid or marginal averages thereof) for each step.

### Appendix A.2. Models 3–8 Studied the Effects of COVID, Exercise Steps, and Maximum Load on the Blood Pressure Parameters at Rest, during Exercise on the Cycloergometer and in the Recovery Phase

The BP parameters included systolic BP (model 3), diastolic BP (model 4), heart rate (model 5), saturation SpO_2_ (model 6), pulse pressure (model 7), and mean BP (model 8) as dependent variables.

The 10 × 2 factorial model design for each dependent variable enabled the examination of the main effects for rest, exercise, and recovery steps (rest (as the reference level), activity for 3, 6, 9 min, activity max, recovery for 3, 6, 9, 12, 15 min) on patients after COVID-19 and the control group along with the interaction effect. An additional load max model variable in the form of a covariate enabled the estimation of the effects for the marginal load max mean.

Formula (A4) for models 3–8 is as follows:

(A4) $$\mu_{ij}=\beta_{0}+\beta_{1}\cdot {step}_{ij}+\beta_{2}\cdot {group}_{ij}+\beta_{3}\cdot {\max\;load}_{ij}+\beta_{4}\cdot {\max\;load}_{ij}\times {step}_{ij}\times {group}_{ij}+u_{0i}$$

where: *β*_3_ is the effect of a 1-Watt increase in maximum load.

## Appendix B. The Estimated Marginal Means (EMMs) for Step and Group Predictors in a Linear Model for All Models Are Shown in Table A1

**Table A1:** The Estimated Marginal Means (EMMs) for Step and Group Predictors in a Linear Model for All Models.

Model	Response Variable	Parameter	Control Group									
			Rest	Activity				Recovery				
				3 min	6 min	9 min	Max	3 min	6 min	9 min	12 min	15 min
Model 1	Load [Watt]	EMMs	-	50.5	-	-	257.5	-	-	-	-	-
		SE	-	6.3	-	-	6.3	-	-	-	-	-
		95% CI	-	[38.1–62.9]	-	-	[245.1–269.9]	-	-	-	-	-
Model 2	O_2_ pulse [mL/beat]	EMMs	6.07	11.4	-	-	17.01	5.16	-	-	-	-
		SE	0.361	0.361	-	-	0.361	0.361	-	-	-	-
		95% CI	[5.36–6.78]	[10.69–12.11]	-	-	[16.30–17.72]	[4.45–5.86]	-	-	-	-
Model 3	SBP [mmHg]	EMMs	123	127	144	163	180	146	126	119	116	114
		SE	1.97	1.97	1.97	1.97	1.97	1.97	1.97	1.97	1.97	1.97
		95% CI	[119–127]	[123–131]	[140–147]	[157–167]	[176–184]	[142–149]	[122–130]	[115–123]	[112–119]	[110–118]
Model 4	DBP [mmHg]	EMMs	78.7	78.7	79.6	81.6	82	79.9	76.6	76.7	76.7	75.9
		SE	1.34	1.34	1.34	1.35	1.34	1.34	1.34	1.34	1.34	1.34
		95% CI	[76.1–81.3]	[76.1–81.3]	[77.0–82.2]	[79.0–84.3]	[79.3–84.6]	[77.3–82.5]	[73.9–79.2]	[74.1–79.3]	[74.1–79.3]	[73.3–78.5]
Model 5	HR [beats/min]	EMMs	85.1	108.8	126.9	153.5	178.9	112.4	102.2	100.6	100.7	97.5
		SE	1.92	1.92	1.92	1.93	1.92	1.92	1.92	1.92	1.92	1.92
		95% CI	[81.4–88.9]	[105.0–112.6]	[123.1–130.6]	[149.7–157.3]	[175.1–182.7]	[108.6–116.2]	[98.4–106.0]	[96.8–104.4]	[97.0–104.5]	[93.7–101.2]
Model 6	Saturation [%]	EMMs	99.38	99.25	99.29	98.94	97.45	99.38	99.3	99.23	99.17	99.14
		SE	0.15	0.15	0.15	0.15	0.15	0.15	0.15	0.15	0.15	0.15
		95% CI	[99.09–99.67]	[98.96–99.55]	[99.00–99.58]	[98.64–99.24]	[97.15–97.74]	[99.09–99.68]	[99.00–99.59]	[98.94–99.52]	[98.88–99.47]	[98.85–99.43]
Model 7	PP [mmHg]	EMMs	44	48.5	63.9	81.1	97.7	65.7	49.6	42	38.9	38.4
		SE	1.51	1.51	1.51	1.54	1.51	1.51	1.51	1.51	1.51	1.51
		95% CI	[41.0–47.0]	[45.5–51.5]	[60.9–66.9]	[78.1–84.1]	[94.8–100.7]	[62.7–68.7]	[46.6–52.4]	[39.0–44.9]	[35.9–41.8]	[35.4–41.4]
Model 8	MBP [mmHg]	EMMs	93.4	94.9	100.9	108.7	114.5	101.8	93.1	90.7	89.7	88.7
		SE	1.4	1.4	1.4	1.42	1.4	1.4	1.4	1.4	1.4	1.4
		95% CI	[90.6–96.1]	[92.1–97.6]	[98.1–103.7]	[105.9–111.5]	[111.8–117.3]	[99.0–104.5]	[90.3–95.9]	[87.9–93.5]	[86.9–92.4]	[85.9–91.5]
**Model**	**Response Variable**	**Parameter**	**COVID Group**									
			**Rest**	**Activity**				**Recovery**				
				**3 min**	**6 min**	**9 min**	**Max**	**3 min**	**6 min**	**9 min**	**12 min**	**15 min**
Model 1	Load [Watt]	EMMs	-	49.6	-	-	223.6	-	-	-	-	-
		SE	-	49.6	-	-	223.6	-	-	-	-	-
		95% CI	-	[38.3–60.9]	-	-	[212.3–234.9]	-	-	-	-	-
Model 2	O_2_ pulse [mL/beat]	EMMs	5.71	10.63	-	-	14.99	4.47	-	-	-	-
		SE	0.329	0.329	-	-	0.329	0.329	-	-	-	-
		95% CI	[5.06–6.35]	[9.98–11.28]	-	-	[14.34–15.63]	[3.82–5.12]	-	-	-	-
Model 3	SBP [mmHg]	EMMs	125	132	148	167	182	149	128	122	122	120
		SE	1.78	1.78	1.78	1.8	1.78	1.78	1.78	1.78	1.78	1.78
		95% CI	[121–128]	[128–135]	[144–151]	[163–170]	[179–186]	[145–152]	[124–131]	[118–125]	[119–126]	[117–124]
Model 4	DBP [mmHg]	EMMs	81.7	80.8	82.2	83.9	83.4	81.5	80	79.7	81.2	80.8
		SE	1.2	1.2	1.2	1.2	1.22	1.2	1.2	1.2	1.2	1.2
		95% CI	[79.3–84.1]	[78.5–83.2]	[79.9–84.6]	[81.5–86.2]	[81.1–85.8]	[79.2–83.9]	[77.6–82.3]	[77.3–82.1]	[78.8–83.5]	[78.5–83.2]
Model 5	HR [beats/min]	EMMs	84.9	111.6	131.6	153.5	177.5	112	103.2	101.8	100.4	99.1
		SE	1.73	1.73	1.73	1.74	1.73	1.73	1.73	1.73	1.73	1.73
		95% CI	[81.5–88.3]	[108.2–115.0]	[128.2–135.0]	[150.0–156.9]	[174.1–180.9]	[108.6–115.4]	[99.8–106.6]	[98.4–105.2]	[97.0–103.8]	[95.7–102.6]
Model 6	Saturation [%]	EMMs	99.41	99.33	99.23	98.83	97.46	99.47	99.26	99.17	99.23	99.17
		SE	0.13	0.13	0.13	0.13	0.13	0.13	0.13	0.13	0.13	0.13
		95% CI	[99.15–99.68]	[99.07–99.60]	[98.96–99.49]	[98.56–99.10]	[97.19–97.72]	[99.20–99.73]	[98.99–99.52]	[98.90–99.43]	[98.96–99.49]	[98.91–99.44]
Model 7	PP [mmHg]	EMMs	43.2	51.2	65.6	82.7	98.9	67.3	47.8	43.3	40.9	39.5
		SE	1.36	1.36	1.36	1.38	1.36	1.36	1.36	1.36	1.36	1.36
		95% CI	[40.5–45.9]	[48.5–53.8]	[63.0–68.3]	[80.0–85.4]	[96.3–101.6]	[64.6–69.9]	[45.1–50.5]	[39.6–44.9]	[38.2–43.6]	[36.9–42.2]
Model 8	MBP [mmHg]	EMMs	96.1	97.9	104.1	111.4	116.4	104	95.9	93.8	94.8	94
		SE	1.27	1.27	1.27	1.28	1.27	1.27	1.27	1.27	1.27	1.27
		95% CI	[93.6–98.6]	[95.4–100.4]	[101.6–106.6]	[108.9–113.9]	[113.9–118.9]	[101.5–106.5]	[93.4–98.4]	[91.3–96.3]	[92.3–97.3]	[91.5–96.5]

Note: DFs: model 1—254, model 2—331, model 3—418, model 4—362, model 5—279, models 6,7—771.2, model 8—351. EMMs for each step in models 3–8 conditioned by a marginal mean maximum load = 239 Watt.

## Appendix C. The Results of the Simple Contrasts Used to Estimate the Effects between the Groups within a Marginal Mean Maximum Load = 239 Watts and the Steps Are Shown in Table A2

**Table A2:** The Results of the Simple Contrasts Used to Estimate the Effects between the Groups within a Marginal mean Maximum load = 239 Watts and the Steps.

Model Nr	Response Variable	Parameter	Contrast Control—COVID									
			Rest	Activity				Recovery				
				3 min	6 min	9 min	Max	3 min	6 min	9 min	12 min	15 min
Model 1	Load [Watt]	estimate	-	0.93	-	-	33.96	-	-	-	-	-
		t.ratio	-	0.11	-	-	3.99	-	-	-	-	-
		*p*	-	0.913	-	-	<0.001	-	-	-	-	-
Model 2	O_2_ pulse [mL/beat]	estimate	0.36	0.77	-	-	2.02	0.69	-	-	-	-
		t.ratio	0.74	1.58	-	-	4.14	1.4	-	-	-	-
		*p*	0.459	0.115	-	-	<0.001	0.161	-	-	-	-
Model 3	SBP [mmHg]	estimate	−2.17	−4.79	−4.38	−3.81	−2.67	−3.23	−1.59	−3.29	−6.46	−6.09
		t.ratio	−0.82	−1.81	−1.65	−1.41	−1.01	−1.22	−0.6	−1.24	−2.44	−2.3
		*p*	0.414	0.072	0.1	0.157	0.315	0.224	0.549	0.215	0.015	0.022
Model 4	DBP [mmHg]	estimate	−2.99	−2.15	−2.66	−2.22	−1.46	−1.66	−3.39	−3.01	−4.44	−4.95
		t.ratio	−1.66	−1.19	−1.48	−1.22	−0.81	−0.92	−1.88	−1.67	−2.47	−2.75
		*p*	0.097	0.233	0.141	0.222	0.418	0.356	0.061	0.096	0.014	0.006
Model 5	HR [beats/min]	estimate	0.25	−2.78	−4.72	0.06	1.44	0.38	−1.01	−1.24	0.3	−1.68
		t.ratio	0.1	−1.08	−1.83	0.02	0.56	0.15	−0.39	−0.48	0.12	−0.65
		*p*	0.922	0.283	0.068	0.981	0.576	0.883	0.695	0.631	0.908	0.516
Model 6	Saturation [%]	estimate	−0.03	−0.08	0.06	0.11	−0.01	−0.08	0.04	0.06	−0.05	−0.03
		t.ratio	−0.16	−0.4	0.3	0.55	−0.05	−0.42	0.2	0.3	−0.27	−0.16
		*p*	0.871	0.691	0.767	0.58	0.961	0.678	0.839	0.763	0.789	0.872
Model 7	PP [mmHg]	estimate	0.82	−2.64	−1.72	−1.5	−1.21	−1.57	1.79	−0.29	−2.02	−1.14
		t.ratio	0.4	−1.3	−0.85	−0.77	−0.59	−0.77	0.88	−0.14	−0.99	−0.56
		*p*	0.686	0.195	0.399	0.44	0.553	0.442	0.379	0.888	0.322	0.577
Model 8	MBP [mmHg]	estimate	−2.72	−3.03	−3.23	−2.75	−1.86	−2.18	−2.79	−3.1	−5.12	−5.33
		t.ratio	−1.44	−1.6	−1.71	−1.44	−0.98	−1.15	−1.47	−1.64	−2.71	−2.82
		*p*	0.152	0.11	0.089	0.151	0.326	0.249	0.142	0.102	0.007	0.005

Note: DFs: model 1—331, model 2—137, model 3—418, model 4—362, model 5—279, models 6,7—771.2, model 8—351. EMMs for each step in models 3–8 conditioned by a marginal mean maximum load = 239 Watt.
